# Outcomes of the Treatment of Fracture Non-union Using Combined Magnetic Field Bone Growth Stimulation: Experiences From a UK Trauma Unit

**DOI:** 10.7759/cureus.25100

**Published:** 2022-05-18

**Authors:** Vusumuzi Sibanda, Fitzgerald Anazor, Jai Relwani, Baljinder S Dhinsa

**Affiliations:** 1 Trauma and Orthopaedics, William Harvey Hospital, Ashford, GBR

**Keywords:** bone stimulators, fracture union, fracture non-union, combined magnetic field, electromagnetic stimulation, bone healing, fracture healing

## Abstract

Introduction: Fracture non-union is a distressing diagnosis for both patients and clinicians. Several methods have been tried to help promote bone healing. Some of the non-operative strategies include the use of pulsed ultrasound and electrical or magnetic bone stimulators. This study aimed at assessing the outcomes of patients treated with combined magnetic field (CMF) bone stimulators.

Methods: All patients with confirmed fracture non-union treated using a CMF bone growth stimulator between May 2019 and December 2021 were included in the study. These were followed up at regular three-month intervals and monitored for signs of clinical and radiological union. The minimum patient follow-up was six months. Our primary outcome measure was union rates following CMF treatment. The secondary outcome measures were time to union and fracture type/configuration in relation to non-union.

Results: A total of 29 patients were included. Of the patients, 52% were female. The average age of the patients was 53.42 years (SD: 17.66 years). Four were excluded because their follow-up period was less than six months. Patients were started on CMF bone growth stimulant treatment between four and 27 months from the initial fracture (mean: 11.56 months). The majority of the patients had tibial shaft (21%), distal femur (17%), ankle (10%) and distal humerus (10%) fractures. The overall success rate was 84% (n=21), with a mean time to union of 6.62 months.

Conclusion: Bone growth stimulators using combined magnetic fields are a viable treatment option for established fracture non-union. They can result in improved outcomes and can avoid risks and costs associated with surgical options to treat non-union. However, more studies need to be conducted to establish the efficacy of these methods conclusively.

## Introduction

The physiology of fracture healing is an intricate and complex process. Bone healing can occur either by direct (primary) or indirect (secondary) methods. Secondary healing involves the formation of a haematoma at the fracture site, an inflammatory phase, generation of callus (fibrocartilaginous and bony) and finally bone remodelling. These stages have considerable overlap [1]. On the other hand, primary bone healing results in the re-establishment of the cortex without callus formation. This occurs in circumstances where there is absolute stability at the fracture site commonly achieved by open reduction and internal fixation using lag screw fixation or compression plating [2].

Several factors influence bone healing, and these can be classified broadly into host, biological and mechanical factors. Host factors include smoking, age, gender, alcohol use, diabetes, steroid use, NSAID use and patient compliance, whilst biological factors include blood supply, infection, degree of soft tissue damage and degree of bone loss. Mechanical factors include fracture configuration, fixation method and degree of immobilisation [3,4]. When adverse factors such as smoking, infection, steroid use and severe soft tissue damage are present, fracture non-union may occur.

Fracture non-union is associated with high morbidity and clinical burden [4]. There is no agreed definition of fracture non-union. Typically, a fracture is said to be in non-union if there is a failure for the bone to unite (clinically and/or radiologically) after 6-9 months [5]. However, this varies with the bone affected, patient age and fracture pattern. Clinical union is usually characterised by an absence of tenderness, bony crepitus and abnormal movement at the fracture site. Radiological union is easier to assess in cases of secondary bone healing with callus formation and is characterised by callus formation in at least three cortices, presence of bridging cortical trabeculae and/or visible endosteal callus formation.

Studies have shown that the rates of fracture non-union range between 5% and 10% of all fractures. In the UK, the costs to the NHS for treating non-union range between £7,000 and £79,000 per person [6,7]. In contrast, a combined magnetic field (CMF) bone stimulator such as the one used for our study will cost £1,425 for the entire duration of the treatment. In addition to the financial implications, non-union can also negatively impact physical and mental health and quality of life [8].

Therefore, it is imperative that several strategies have been tried to stimulate bone healing, which can be employed in circumstances of fracture non-union. One such method is using electrical and magnetic fields (EMF) to encourage bone healing. The combined magnetic field (CMF) treatment employs a combination of direct and alternating current to generate a sinusoidal wave pattern of electrical stimulation [9]. These are non-invasive devices whose exact mechanism of action is not clearly understood, but they have been shown to upgrade bone-promoting growth factors such as insulin-like growth factor-II (IGF-II), calcitonin, interleukin-2 (IL-2), calmodulin and insulin [10]. Their efficacy remains a subject of debate. The National Institute for Health and Care Excellence (NICE) guidance state that the evidence of their efficacy is inadequate in quality, and therefore, the procedure should be used with special arrangements for clinical governance, consent and audit or research [11].

This study sought to assess the outcomes of patients with established fracture non-union of distal small bones and long bones treated with combined magnetic field (CMF) bone stimulators regardless of the fracture type. Our primary outcome measure was union rates following CMF treatment. The secondary outcome measures were time to union and fracture type/configuration in relation to non-union.

## Materials and methods

Our study was registered as a service evaluation with the Clinical Audit Department in the East Kent Hospitals University NHS Foundation Trust, and study approval was obtained (approval number: RN783477). Only anonymised patient data was collected, and each patient gave consent for CMF treatment as per standard service procedure. We only followed up with those who had CMF treatment after it had been commenced by their treating clinician. We did not contact patients at home to monitor compliance but allowed them to follow their treating consultant's recommendation as per the manufacturer's guidance. We did not alter any routine treatment pathway for any patient or participate in CMF treatment recommendations for any patient.

The study was a retrospective review of patients treated with combined magnetic field (CMF) bone stimulators over two and half years, from May 2019 to December 2021. Only patients who were deemed to have clinical and/or radiological evidence of non-union by a consultant orthopaedic surgeon were recruited. The patients were taken from all the orthopaedic subspecialties, including upper limb, lower limb and foot and ankle. The minimum time from the initial fracture or fixation to recruitment for CMF treatment was four months.

The patients were prescribed the CMF device (DJO Global, UK). The devices are battery-powered and non-invasive with a shape designed to act on the part of the body that they are prescribed for. The patients were instructed to undergo daily 30-minute treatment sessions at home for the entire duration of their treatment. They were followed up at regular three-month intervals and monitored for signs of clinical and radiological union. The minimum patient follow-up for this study was six months.

Details of patient characteristics such as age, comorbidities and smoking status were collected and analysed. Information on fracture characteristics, including the type of fracture, initial fracture management and subsequent treatment in the event of failure of union, was also collected. At the end of the treatment period, the patients were then discharged from the outpatient clinic. Patients who were deemed to have failed treatment were offered other treatment modalities for their fracture non-union.

The data was entered into an Excel spreadsheet (Microsoft Corporation, Redmond, Washington, USA) and analysed.

## Results

A total of 29 patients were recruited in the 32 months from May 2019 to December 2021. Of these, 14 (48%) were male, and 15 (52%) were female. Four patients were excluded because their follow-up period was less than six months. This left 25 patients who were then studied and analysed.

The average age of the patients was 53.42 years (SD: 17.66 years). On analysis of risk factors, six (24%) had a smoking history, and 10 (40%) had a significant alcohol-taking history. 

The patients were started on combined magnetic field (CMF) bone stimulator treatment between four and 27 months from the initial fracture (mean: 11.56). They were only started on treatment following assessment by an orthopaedic consultant and deemed to be in non-union.

**Figure 1:**
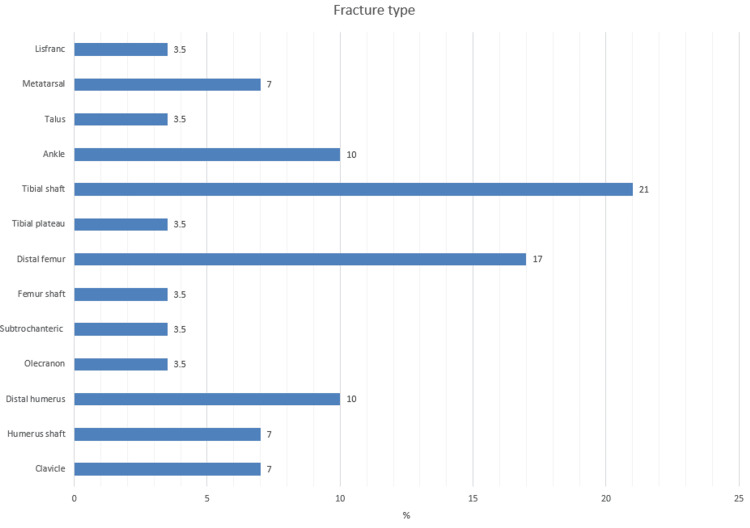
Types of fracture non-unions and their frequency

The majority of the patients had tibial shaft (21%), distal femur (17%), ankle (10%) and distal humerus (10%) fractures. Other patients had metatarsal (7%), clavicle (7%), humeral shaft (7%), talus (3.5%), tibial plateau (3.5%), femoral shaft (3.5%), subtrochanteric (3.5%), Lisfranc (3.5%) and olecranon (3.5%) fractures (Figure 1).

Of the patients, 17 (68%) had surgery as the initial treatment for their fractures, whilst eight (32%) had been managed conservatively.

**Figure 2:**
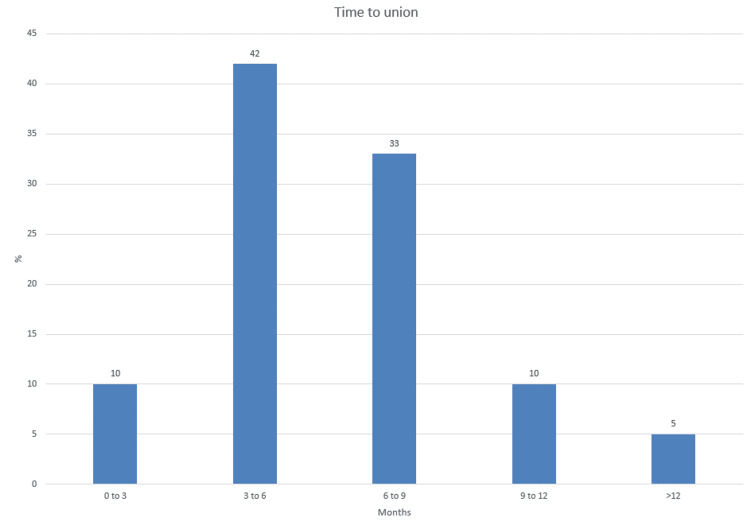
Time to union in months

The overall success rate was 84% (n=21), with an average time to union of 6.62 months. A t-test of the data has shown a p-value of 0.006 (level of significance: p<0.01), which has confirmed that the data is statistically significant for the success rate of the bone stimulator treatment. Of the patients, 10% achieved union within three months of starting treatment, whilst 42% united in 3-6 months, 33% in 6-9 months, 10% in 9-12 months and 5% in >12 months (Figure 2).

Four of the patients did not achieve union after being followed up for six, 11 and 14 months. The patients had diagnoses of midshaft clavicle, distal femur, tibial plateau and Lisfranc fractures, respectively. The midshaft clavicle and distal femur fractures had been initially treated with open reduction and internal fixation, whilst the Lisfranc fracture had a fusion in the first instance. The tibial plateau had been treated conservatively initially. They went on to receive subsequent treatment. The patients with clavicle and distal femur fractures had bone grafting, whilst the patient with tibial plateau fracture had a total knee replacement, and the patient with Lisfranc fracture had debridement and revision of fusion. All four patients went on to achieve union after the surgical interventions had been done.

## Discussion

In our study, 52% of the patients were female. Other studies show a mixed picture for the incidence of fracture non-union based on sex. For example, a large Scottish registry study by Mills and Simpson in 2013 showed that 56% were male, whereas Zura et al. in 2016 had 58% of the cases in male patients [6,7]. Both these studies had large sample sizes; therefore, their findings can easily be generalised to the general population.

Our series had more patients with lower limb fracture non-unions (72.5%) compared to upper limb fracture non-unions (27.5%). This is different from the epidemiological study from the Scottish registry by Mills and Simpson (2013) who had 60% of the non-unions identified in upper limb fractures and 40% in lower limb fractures [7]. This difference is likely due to the differences in sample size as our series had a relatively lower number of patients and is not an epidemiological study. However, the lower limb non-unions in their study were more common in the tibia, which is similar to our findings. Their study also showed that non-union has a widespread distribution in the appendicular skeleton affecting multiple sites, which is similar to our findings.

The findings from our retrospective study show that the majority of the fracture non-unions went on to achieve union within nine months of CMF treatment. This is comparable with the data from the double-blind, randomised control trial by Linovitz et al., who had a 64% union rate within nine months following CMF treatment for spinal fusions compared to 43% for placebo devices with a statistically significant result [12]. However, a prospective observational study by Phillips et al. showed an overall union rate of 45% at nine months following CMF treatment with a 76% success rate for the tibial fracture subtype [13]. The relatively wide variations in success rates can be attributed to the differences in fracture type and the effects of confounding variables on fracture healing, such as smoking and medical comorbidities.

A meta-analysis by Mollon et al. (2008) favoured electrical stimulation treatment in four out of five studies where treatment was employed for fracture non-union. However, the studies analysed mainly involved the use of pulsed electromagnetic therapy and capacitance coupling, which are other methods of electrical stimulation in fracture healing with slightly different physics principles [14].

On the other hand, a multicentre, double-blind randomized control trial comparing pulsed electromagnetic field (PEF) stimulation versus placebo by Adie et al. (2011) showed no significant difference in union rates or prevention of secondary surgical intervention to treat non-union [15]. This study involved the use of PEF stimulation, which is similar to CMF. However, their results could be due to the slightly different properties of CMF and PEF. Moreover, in their study, they offered PEF stimulation to acute fractures, whereas in our cohort, we offered bone stimulators to patients with established non-union. The varying results from some of the studies highlighted above buttresses the continuous debate about the use of electromagnetic field stimulation to promote fracture healing. More well-designed, randomized control studies and meta-analyses will be needed in the future to resolve this debate.

Our study had some strengths, including a relatively long follow-up period. The minimum follow-up period was six months, with some patients being followed up beyond 12-18 months. We also included a wide variety of fracture types, including distal small bones and long bones. We also ensured that the assessment of union was done by a consultant orthopaedic surgeon, and we used a fixed daily treatment duration per patient. This ensured the standardisation of treatment for each patient.

There are some limitations to our study. It was a retrospective, observational study with no randomisation/blinding, and we cannot confidently exclude the possibility that some of these fractures might have healed due to alterations in other patient biological factors. Some fractures might have gone on to unite given a longer duration. Also, we have not analysed the effect of confounding factors such as smoking, medical comorbidities, steroid use or soft tissue compromise as these could have a complex interplay on fracture healing, and doing so with our relatively small sample size may not yield solid statistical correlations. We have also not monitored patients at home to ensure strict compliance to the daily 30-minute treatment regimen. Our study's average time to starting CMF was four months, which is a bit early compared to some papers.

There is a possibility of inter-observer variation in assessing radiological union as we relied on the assessment of union by an orthopaedic surgeon. This could have been strengthened via multiple assessments by at least two clinicians, including a radiology consultant. In daily practice, non-union in the clinical setting is usually diagnosed by the treating orthopaedic surgeons who are well-trained to do this. However, in our study, each patient's imaging was reviewed by the senior authors (JR and BSD) to confirm agreement of diagnosis of non-union.

The relatively small sample size in our study is another limitation. However, this study aims not to define what treatment options conclusively work for non-union but to show the findings from our single-centre patient cohort.

## Conclusions

In summary, our series had a relatively small number of patients and is designed to show our single-centre experience with the use of CMF. We have shown that CMF is successful in our series of patients, but we cannot draw widespread generalisations or make strong recommendations based on these results.

More level 1 studies are needed to provide quality evidence on the role of combined magnetic field treatment in fracture healing in order to convince treating clinicians and patients that these are more efficacious than placebo for treating fracture non-union. We hope that our study will help stimulate more research in this direction as this non-invasive treatment modality has the potential to reduce cost and risk to well-selected patients, thus possibly leading to a reduction in the risks associated with repeated surgical interventions for fracture non-union.
